# Supplementary material for: Clusters of diet, physical activity, screen-time and sleep among adolescents and associations with 3-year change in indicators of adiposity
Source: PLoS One. 2024 Dec 23;19(12):e0316186. doi: 10.1371/journal.pone.0316186 (PMC11666017; doi:10.1371/journal.pone.0316186)
Supplement: S1 Table — Abbreviations: OECD, organisation for economic co-operation and development; NVQ, national vocational qualification. (DOCX) [file pone.0316186.s001.docx]

|  | **Excluded** | **Included** | **Overall**  **(N=9,096)** | **P value** |
| --- | --- | --- | --- | --- |
| **Sex** |  |  |  |  |
| Boys | 2,981  (49.44%) | 1,458  (47.54%) | 4,439  (48.80%) | 0.086 |
| Girls | 3,048  (50.56%) | 1,609  (52.46%) | 4,657  (51.20%) |  |
| **Ethnicity** |  |  |  |  |
| White | 4,525  (75.05%) | 2,598  (84.71%) | 7,123  (78.31%) | **< 0.001** |
| Other | 1,504  (24.95%) | 469  (15.29%) | 1,973  (21.69%) |  |
| **OECD weekly family income** | 398.13 (261.23 - 540.26) | 453.24 (312.37 - 580.31) | 417.97 (275.90 - 556.71) | **< 0.001** |
| **NVQ Highest Level** |  |  |  |  |
| Low | 2,501  (41.72%) | 1,256  (40.95%) | 3,757  (41.46%) | **< 0.001** |
| High | 2,943  (49.09%) | 1,650  (53.80%) | 4,593  (50.68%) |  |
| Other qualifications | 551  (9.19%) | 161  (5.25%) | 712  (7.86%) |  |
