# Supplementary material for: Clusters of diet, physical activity, screen-time and sleep among adolescents and associations with 3-year change in indicators of adiposity
Source: PLoS One. 2024 Dec 23;19(12):e0316186. doi: 10.1371/journal.pone.0316186 (PMC11666017; doi:10.1371/journal.pone.0316186)
Supplement: S2 Table — Abbreviations: OECD, organisation for economic co-operation and development; NVQ, national vocational qualification; hrs, hours; BMI, body mass index. *>60 minutes of moderate to vigorous physical activity per day. (DOCX) [file pone.0316186.s002.docx]

| **Cluster** | **Cluster 1**  **Healthy Cluster**  N= 925  (30.18%) | **Cluster 2**  **Mixed Cluster**  N= 1346  (43.92%) | **Cluster 3**  **Unhealthy Cluster**  N= 794  (25.91%) | ***P* value** |
| --- | --- | --- | --- | --- |
| **Gender** |  |  |  | 0.001 |
| Boys | 408  (44.11%) | 628  (46.66%) | 421  (53.02%) |  |
| Girls | 517  (55.89%) | 718  (53.34%) | 373  (46.98%) |  |
| **Ethnicity** |  |  |  | 0.005 |
| White | 813  (87.89%) | 1,124  (83.51%) | 659  (83.00%) |  |
| Other | 112  (12.11%) | 222  (16.49%) | 135  (17.00%) |  |
| **Puberty** |  |  |  | 0.189 |
| Pre/early puberty | 361  (39.15%) | 482  (35.94%) | 310  (39.19%) |  |
| Post/late pubertal | 561  (60.85%) | 859  (64.06%) | 481  (60.81%) |  |
| **OECD weekly family income** | 515 (166) | 442 (171) | 394 (164) | <0.001 |
| **NVQ Highest Level (all sweeps)** |  |  |  | <0.001 |
| Low | 282  (30.49%) | 580  (43.09%) | 393  (49.50%) |  |
| High | 622  (67.24%) | 692  (51.41%) | 335  (42.19%) |  |
| Other qualifications | 21  (2.27%) | 74  (5.50%) | 66  (8.31%) |  |
| **Physical activity** |  |  |  | <0.001 |
| Not meeting the guidelines* | 433  (46.81%) | 808  (60.03%) | 529  (66.62%) |  |
| Meeting the guidelines* | 492  (53.19%) | 538  (39.97%) | 265  (33.38%) |  |
| **Screen time (hrs/weekday)** |  |  |  |  |
| **Watching TV / videos / computer** |  |  |  | <0.001 |
| < 2 | 532  (57.51%) | 405  (30.09%) | 109  (13.73%) |  |
| > 2 | 393  (42.49%) | 941  (69.91%) | 685  (86.27%) |  |
| **Playing electronic games** |  |  |  | <0.001 |
| < 2 | 795  (85.95%) | 828  (61.52%) | 274  (34.51%) |  |
| > 2 | 130  (14.05%) | 518  (38.48%) | 520  (65.49%) |  |
| **Social networking sites** |  |  |  | <0.001 |
| < 2 | 709  (76.65%) | 723  (53.71%) | 222  (27.96%) |  |
| > 2 | 216  (23.35%) | 623  (46.29%) | 572  (72.04%) |  |
| **Sleep duration (hrs/day)** |  |  |  | <0.001 |
| < 9 | 315  (34.05%) | 581  (43.16%) | 447  (56.30%) |  |
| > 9 | 610  (65.95%) | 765  (56.84%) | 347  (43.70%) |  |
| **Dietary intake** |  |  |  |  |
| **Sweetened beverages (frequency/week)** |  |  |  | <0.001 |
| < 3 | 804  (86.92%) | 894  (66.42%) | 179  (22.54%) |  |
| > 3 | 121  (13.08%) | 452  (33.58%) | 615  (77.46%) |  |
| **At least 2 portions of fruit per day** |  |  |  | <0.001 |
| Never / some days | 214  (23.14%) | 1,044  (77.56%) | 774  (97.48%) |  |
| Every day | 711  (76.86%) | 302  (22.44%) | 20  (2.52%) |  |
| **At least 2 portions of vegetables per day** |  |  |  | <0.001 |
| Never / some days | 129  (13.95%) | 933  (69.32%) | 751  (94.58%) |  |
| Every day | 796  (86.05%) | 413  (30.68%) | 43  (5.42%) |  |
| **Indicators of adiposity at age 14** |  |  |  |  |
| Body fat % at 14 | 21.1 (8.6) | 21.94 (8.9) | 22.1 (9.2) | 0.0481 |
| BMI z-score at 14 | 0.51 (1.1) | 0.60 (1.2) | 0.71 (1.2) | 0.0012 |
| BMI category |  |  |  | 0.014 |
| Normal/Under-weight | 734  (79.35%) | 1,042  (77.41%) | 579  (72.92%) |  |
| Overweight | 149  (16.11%) | 222  (16.49%) | 155  (19.52%) |  |
| Obese | 42  (4.54%) | 82  (6.09%) | 60  (7.56%) |  |
|  |  | 4 (1 - 8) | 5 (2 - 10) | 0.0001 |
| **Indicators of adiposity at age 17** |  |  |  |  |
| Body fat % at 17 | 21.8 (9.6) | 22.2 (10.1) | 22.2 (10.6) | 0.6609 |
| BMI z-score at 17 | 1.1 (1.0) | 1.1 (1.1) | 1.3 (1.1) | 0.0102 |
| BMI category 17 |  |  |  | <0.001 |
| Normal/Under-weight | 697  (75.35%) | 984  (73.11%) | 538  (67.76%) |  |
| Overweight | 169  (18.27%) | 227  (16.86%) | 164  (20.65%) |  |
| Obese | 59  (6.38%) | 135  (10.03%) | 92  (11.59%) |  |
|  | 6 (4 -10) | 6 (4 - 10) | 7 (4 - 11) | 0.0012 |
