# Supplementary figures and images for: Clusters of diet, physical activity, screen-time and sleep among adolescents and associations with 3-year change in indicators of adiposity
Source: PLoS One. 2024 Dec 23;19(12):e0316186. doi: 10.1371/journal.pone.0316186 (PMC11666017; doi:10.1371/journal.pone.0316186)

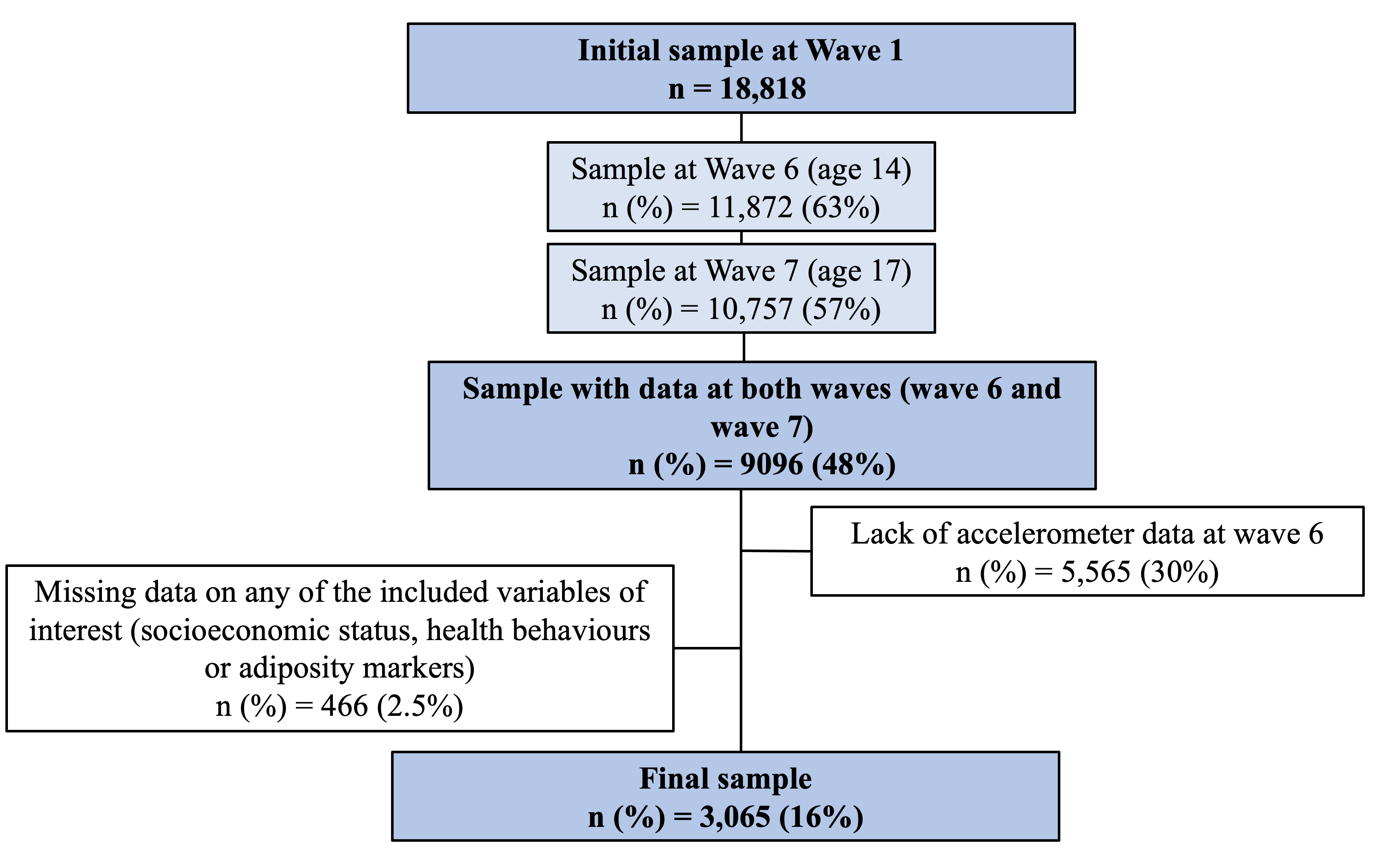

Supplement: S1 Fig — (DOCX) [file pone.0316186.s003.docx]
