# Supplementary material for: Clusters of diet, physical activity, screen-time and sleep among adolescents and associations with 3-year change in indicators of adiposity
Source: PLoS One. 2024 Dec 23;19(12):e0316186. doi: 10.1371/journal.pone.0316186 (PMC11666017; doi:10.1371/journal.pone.0316186)
Supplement: Attachment — Submitted filename: PONE-D-24-34337_reviewer.pdf [file pone.0316186.s004.pdf]

PLOS ONE

Clusters of Diet, Physical Activity, Screen-Time, and Sleep Among Adolescents and Associations with 3-Year Change in Indicators of Adiposity

--Manuscript Draft--

**Manuscript Number:**	PONE-D-24-34337
**Article Type:**	Research Article
**Full Title:**	Clusters of Diet, Physical Activity, Screen-Time, and Sleep Among Adolescents and Associations with 3-Year Change in Indicators of Adiposity
**Short Title:**	Clusters of health behaviours and 3-year change in indicators of adiposity among adolescents
**Corresponding Author:**	Noura Alosaimi<br>Loughborough University School of Sport Exercise and Health Sciences<br>Loughborough, UNITED KINGDOM OF GREAT BRITAIN AND NORTHERN IRELAND
**Keywords:**	Clustering of unhealthy behaviours, sedentary behaviours, dietary behaviours, obesity, adolescents, accelerometer, BMI, percent body fat, MVPA, sleep
**Abstract:**	Background: Clusters of health behaviours could impact changes in adiposity among adolescents over time. This study examines the clustering of screen time, physical activity, dietary behaviours and sleep, and the associations with 3-year changes in indicators of adiposity.<br><br>Methods: Data from the UK's Millennium Cohort Study were utilised when participants were aged 14 and 17 years respectively. At age 14, demographics, screen time, dietary behaviours and sleep duration were measured via self-report, and physical activity using wrist worn accelerometers. Height, weight, and percent body fat were measured at age 14 and 17 years. Behavioural clusters were determined using k-means clustering analysis, and associations with change in indicators of adiposity between age 14 and 17 years were examined using multivariate regression models.<br><br>Results: Three clusters were identified at age 14, a 'healthy cluster', a 'mixed cluster', and an 'unhealthy cluster' in the analytical sample of 3,065 participants (52.5% girls). The 'unhealthy' cluster was the most prevalent cluster among boys (53%), while the 'healthy cluster' was most prevalent among girls (55.9%). Adolescents in healthy clusters had a lower BMI z-score and percent body fat at age 14 compared to those in the unhealthy and mixed clusters, and maintained lower scores at age 17. Boys in the mixed and unhealthy clusters at 14 years had a lower change in BMI z-score between 14 and 17 compared to boys in the healthy cluster.<br><br>Conclusion: Adolescents in the healthy cluster had lower BMI z-scores and percent body fat at age 14 years than those in the unhealthy cluster, and they maintained this lower level at age 17. Given the upward trend in BMI during this period, this maintenance could be interpreted as a positive outcome. Further prospective research is needed to better understand these associations as well as research examining the stability of cluster membership over time.
**Order of Authors:**	Noura Alosaimi
	Lauren B Sherar, Professor
	Paula Griffiths, Professor
	Mark Hamer, Professor
	Natalie Pearson, Doctor
**Additional Information:**	
**Question**	**Response**
**Financial Disclosure**	The author(s) received no specific funding for this work.

Powered by Editorial Manager® and ProduXion Manager® from Aries Systems Corporation

Enter a financial disclosure statement that describes the sources of funding for the work included in this submission. Review the submission guidelines for detailed requirements. View published research articles from *PLOS ONE* for specific examples.<br><br>This statement is required for submission and **will appear in the published article** if the submission is accepted. Please make sure it is accurate.<br><br>**Funded studies**<br>Enter a statement with the following details:<br>• Initials of the authors who received each award<br>• Grant numbers awarded to each author<br>• The full name of each funder<br>• URL of each funder website<br>• Did the sponsors or funders play any role in the study design, data collection and analysis, decision to publish, or preparation of the manuscript?<br><br>Did you receive funding for this work?	
**Competing Interests**<br><br>Use the instructions below to enter a competing interest statement for this submission. On behalf of all authors, disclose any competing interests that could be perceived to bias this work—acknowledging all financial support and any other relevant financial or non-financial competing interests.<br><br>This statement is required for submission and **will appear in the published article** if the submission is accepted. Please make sure it is accurate and that any funding sources listed in your Funding Information later in the submission form are also declared in your Financial Disclosure statement.<br><br>View published research articles from	The authors have declared that no competing interests exist.

PLOS ONE for specific examples.<br><br>**NO authors have competing interests**<br><br>Enter: *The authors have declared that no competing interests exist.*<br><br>**Authors with competing interests**<br><br>Enter competing interest details beginning with this statement:<br><br>*I have read the journal's policy and the authors of this manuscript have the following competing interests: [insert competing interests here]*<br><br>* typeset	
**Ethics Statement**<br><br>Enter an ethics statement for this submission. This statement is required if the study involved:<br><br>• Human participants<br>• Human specimens or tissue<br>• Vertebrate animals or cephalopods<br>• Vertebrate embryos or tissues<br>• Field research<br><br>Write "N/A" if the submission does not require an ethics statement.<br><br>General guidance is provided below. Consult the submission guidelines for detailed instructions. **Make sure that all information entered here is included in the Methods section of the manuscript.**	Ethical approval has been granted for all MCS surveys via the National Health Service Research Ethics Committee system. This ensure that all data is collected in line with ethical guidelines, including informed consent, confidentiality, and participant welfare. Written consent for data collection was obtained from parents during the early waves when cohort members were under the age of 16, and as participants grew older, their own consent was required for continued involvement. Also, ethical issues extend to secondary data analysis, where researchers must adhere to guidelines for the appropriate and responsible use of data. The MCS data can be obtained from the UK Data Service. After user and project registration with the UK Data Service, access to the MCS data was approved, and the datasets from sweeps 6 and 7 were downloaded onto an encrypted personal computer in anonymised STATA file format on November 12, 2020. Since the downloaded data were publicly available and fully anonymised, containing no personally identifiable information, no additional ethical approval was required for the current study.

**Format for specific study types**<br><br>**Human Subject Research (involving human participants and/or tissue)**<br>• Give the name of the institutional review board or ethics committee that approved the study<br>• Include the approval number and/or a statement indicating approval of this research<br>• Indicate the form of consent obtained (written/oral) or the reason that consent was not obtained (e.g. the data were analyzed anonymously)<br><br>**Animal Research (involving vertebrate animals, embryos or tissues)**<br>• Provide the name of the Institutional Animal Care and Use Committee (IACUC) or other relevant ethics board that reviewed the study protocol, and indicate whether they approved this research or granted a formal waiver of ethical approval<br>• Include an approval number if one was obtained<br>• If the study involved *non-human primates*, add *additional details* about animal welfare and steps taken to ameliorate suffering<br>• If anesthesia, euthanasia, or any kind of animal sacrifice is part of the study, include briefly which substances and/or methods were applied<br><br>**Field Research**<br><br>Include the following details if this study involves the collection of plant, animal, or other materials from a natural setting:<br>• Field permit number<br>• Name of the institution or relevant body that granted permission	
**Data Availability**<br><br>Authors are required to make all data underlying the findings described fully available, without restriction, and from the time of publication. PLOS allows rare exceptions to address legal and ethical concerns. See the PLOS Data Policy and FAQ for detailed information.	Yes - all data are fully available without restriction

A Data Availability Statement describing where the data can be found is required at submission. Your answers to this question constitute the Data Availability Statement and **will be published in the article**, if accepted.<br><br>**Important:** Stating 'data available on request from the author' is not sufficient. If your data are only available upon request, select 'No' for the first question and explain your exceptional situation in the text box.<br><br>Do the authors confirm that all data underlying the findings described in their manuscript are fully available without restriction?	
**Describe where the data may be found in full sentences. If you are copying our sample text, replace any instances of XXX with the appropriate details.**<br><br>• If the data are **held or will be held in a public repository**, include URLs, accession numbers or DOIs. If this information will only be available after acceptance, indicate this by ticking the box below. For example: *All XXX files are available from the XXX database (accession number(s) XXX, XXX.)*.<br>• If the data are all contained **within the manuscript and/or Supporting Information files**, enter the following: *All relevant data are within the manuscript and its Supporting Information files.*<br>• If neither of these applies but you are able to provide **details of access elsewhere**, with or without limitations, please do so. For example:<br><br>*Data cannot be shared publicly because of [XXX]. Data are available from the XXX Institutional Data Access / Ethics Committee (contact via XXX) for researchers who meet the criteria for access to confidential data.*<br><br>*The data underlying the results presented in the study are available from (include the name of the third party*	All relevant data are within the manuscript and its Supporting Information files.

and contact information or URL).<br>• This text is appropriate if the data are owned by a third party and authors do not have permission to share the data.<br><br>* typeset	
Additional data availability information:	

Powered by Editorial Manager® and ProduXion Manager® from Aries Systems Corporation

Manuscript

Click here to access/download;Manuscript;Final Draft for Submission.docx

Original research article

Clusters of diet, physical activity, screen-time and sleep among adolescents and associations with 3-year change in indicators of adiposity

Noura Alosaimi[1], Lauren B Sherar[1], Paula Griffiths[1], Mark Hamer[2], Natalie Pearson[1]

[1]School of Sport, Exercise & Health Sciences, Loughborough University, UK; [2]Faculty of Medical Sciences, University College London (UCL), UK.

Corresponding author:

Mrs Noura Alosaimi

School of Sport, Exercise & Health Sciences

Loughborough University

LE11 3TU

Email: n.n.alosaimi@lboro.ac.uk

Abstract

Background: Clusters of health behaviours could impact changes in adiposity among adolescents over time. This study examines the clustering of screen time, physical activity, dietary behaviours and sleep, and the associations with 3-year changes in indicators of adiposity.

Methods: Data from the UK's Millennium Cohort Study were utilised when participants were aged 14 and 17 years respectively. At age 14, demographics, screen time, dietary behaviours and sleep duration were measured via self-report, and physical activity using wrist worn accelerometers. Height, weight, and percent body fat were measured at age 14 and 17 years. Behavioural clusters were determined using k-means clustering analysis, and associations with change in indicators of adiposity between age 14 and 17 years were examined using multivariate regression models.

Results: Three clusters were identified at age 14, a 'healthy cluster', a 'mixed cluster', and an 'unhealthy cluster' in the analytical sample of 3,065 participants (52.5% girls). The 'unhealthy' cluster was the most prevalent cluster among boys (53%), while the 'healthy cluster' was most prevalent among girls (55.9%). Adolescents in healthy clusters had a lower BMI z-score and percent body fat at age 14 compared to those in the unhealthy and mixed clusters, and maintained lower scores at age 17. Boys in the mixed and unhealthy clusters at 14 years had a lower change in BMI z-score between 14 and 17 compared to boys in the healthy cluster.

Conclusion: Adolescents in the healthy cluster had lower BMI z-scores and percent body fat at age 14 years than those in the unhealthy cluster, and they maintained this lower level at age

17. Given the upward trend in BMI during this period, this maintenance could be interpreted as a positive outcome. Further prospective research is needed to better understand these associations as well as research examining the stability of cluster membership over time.

Background

Diet, physical activity, sedentary behaviour and sleep are important modifiable contributors to obesity and its associated comorbidities[1]. Recent evidence suggests that only 18% of adolescents in the UK consume the recommended five portions of fruits and vegetables daily[2], and less than 10% meet the recommendations for sleep, screen-time, and physical activity simultaneously[3]. Health behaviours established during adolescence often become deeply ingrained and can persist into adulthood, shaping long-term health behaviours[4]. Therefore, adolescence appears to be a critical period warranting public health efforts to improve health behaviours.

Evidence suggests that poor dietary behaviours, low levels of physical activity, high levels of sedentary behaviours and poor sleep tend to cluster in young people[5,6]. Each of these health behaviours individually is known to contribute to adiposity in youth[7], but together may have synergistic effects leading to poor health[5,8]. Methods that categorise young people into distinct clusters based on their lifestyle behaviours are becoming increasingly popular for investigating the complex relationships between these behaviours and health outcomes. The prevalence of clusters of unfavourable health behaviours has been shown to increase from 29% in children aged 2–5 years to 73.9% among 16–19-year-olds, with the combination of excessive screen time and poor dietary habits emerging as the most frequent, increasing in prevalence from 14.4% in young children to 45.3% in older adolescents[9]. Whereas the prevalence of clusters of favourable health behaviours (i.e. low sedentary behaviours, high moderate to vigorous physical activity (MVPA) and diet quality) was only 18% in a European study of adolescents[10].

Most recent studies examining the associations between clusters of health behaviours and adiposity have been cross-sectional[11–13]. For example, clusters of unfavourable health behaviours have been associated cross-sectionally with increased body mass index (BMI) in adolescent girls and with higher odds of increased waist circumference and percent body fat in adolescent boys[14]. A recent systematic review synthesised the evidence on the prevalence of clustering of physical activity, sedentary behaviours, and diet among children, adolescents, and young adults and their associations with physical and mental health indicators, and found that individuals in both unhealthy and mixed clusters had higher adiposity[15]. The review also highlighted a scarcity of longitudinal studies and few studies that had included sleep in the analysis of clustering of behaviours. Furthermore, there was a noticeable gap in research examining clustering of health behaviours and associated health outcomes in groups of older adolescents[16]. Although current research on the clustering of such lifestyle behaviours has increased[17–21], little is known about in whom and how health behaviours cluster among adolescents, and whether clusters of health behaviours are associated with change in weight and percent body fat during later adolescence. During this critical period of rapid physical growth and development, the lifestyle behaviours established can have long-lasting effects on health outcomes in adulthood[4]. Therefore, interventions targeting healthy behaviours during later adolescence can be particularly impactful in promoting long-term health and well-being.

The current study is original because it examines the clustering of health behaviours among adolescents, and the associations between clusters and change in adiposity in a large sample of adolescents from the UK. This is significant as it reveals how different health behaviours affect adiposity, informing targeted interventions to reduce adolescent obesity. The aims of this study were to (1) identify clusters of lifestyle behaviours based on fruits and vegetables and sugar sweetened beverages (SSB) intake, MVPA, screen-time and sleep

duration; (2) examine the associations between health behaviour clusters and change in (a) body mass index (BMI) z-score, (b) weight status, (c) percent body fat.

Methods

Sample and Data Collection

This study uses data from Millennium Cohort Study (MCS)[22]. The MCS is a nationally representative longitudinal cohort study of 19,243 babies born around the millennium (Sep 2000 – Jan 2002) in the four home countries of the United Kingdom. The MCS was designed to be multidisciplinary, examining a wide range of factors that influence the health and well-being of children and their families. Information from the main carers (mainly mothers) was first collected (i.e. weekly household income, highest maternal academic qualification level, and child's birthweight and gender) when babies were around 9 months of age and they have since been followed up at age 3, 5, 7, 11, 14, 17, and 23 years. The present study used data from sweeps MCS6 (when children were aged 14 years: n=11,872), primarily due to the availability of comprehensive measures for this research objective, and MCS7 when children were aged 17 years (n=10,757).

Measures

All measures of health behaviours were assessed at age 14 years. Indicators of adiposity were assessed at age 14 and 17 years.

Physical Activity

Physical activity was assessed using wrist worn triaxial GENEActiv accelerometers [23] (Activinsights Ltd, Kimbolton, UK). Full details on the accelerometer data collection protocol have been described elsewhere[23]. In brief, participants were instructed to wear the accelerometer on their non-dominant wrist for two specified full days, one weekday and one weekend day (randomly selected at the time of the interviews). Data were downloaded using GENEActiv software and processed using the GGIR package in R, which includes autocalibration and non-wear detection functions[24]. The data were collected in 5-second epochs and included in the present analyses if participants had ≥ 10 hours of valid wear for both days. Euclidean Norm Minus One (ENMO), a measure of mean acceleration over a 24-h period, was used to estimate the overall physical activity[23]. Duration of MVPA was calculated as the time spent with 80% bout criterion of ≥ 100 ENMO for at least 1 minute[25]. Adolescents were classified into two groups: those who achieved a daily average of 60 minutes of MVPA, and those who did not[26].

Screen-Time

Participants reported how much time in hours that they spent TV viewing, playing video games, and on social networking during a typical weekday. For the present analyses, each screen-time variable was categorised as < 2 hours and > 2 hours[27].

Dietary behaviours

Participants reported how often they ate at least 2 portions of fruit per day, 2 portions of vegetables per day, and how often they drank SSB during a week (i.e., once a day, 3-6 days a week, etc). For the present analyses, fruit and vegetable consumption respectively, were

dichotomised into 'Never/Some days' and 'Every day'. SSB consumption was dichotomised into 'more than 3 times per week' and 'less than 3 times per week'[28].

Sleep

Participants were asked about sleep, and selected sleep-onset time for both school and non-school days (i.e. weekends) from: “Before 9pm”/ “9–9:59pm”/ “10–10:59pm”/ “11–11:59pm”/ “After midnight”, and on wake-up wake-up time for school days and non-school days from: “Before 6am”/ “6–6:59am”/ “7–7:59am”/ “8–8:59am”/ “After 9am” and “Before 8am”/ “8–8:59am”/ “9–9:59am”/ “10–10:59am”/ “11–11:59am”/ “After midday”, respectively. Average sleep onset times were calculated using a weekday-to-weekend weighting ratio of 5:2 and grouped into four categories: Before 10pm (reference group), 10–10:59pm, 11–11:59pm, and after midnight. Sleep onset times were combined with usual wake-up times on both school days and non-school days to calculate average sleep durations. Sleep duration was calculated as the time between the category mid-points for sleep onset and wake time. These durations were weighted in a ratio of 5:2 for weekdays to weekends and were categorised into two groups: < 9 hours and > 9 hours.

Adiposity

Data on three indicators of adiposity (BMI, weight status and percent body fat) were collected during home visits conducted by trained researchers at ages 14 and 17 years. Height was measured using Leicester Height Measure Stadiometers (Seca, Birmingham, UK) to the nearest 0.1 cm. Weight and percentage body fat were assessed using calibrated Tanita BF-522W scales (Tanita UK, Yiewsley, Middlesex, UK). For ages 14 and 17, BMI was calculated by dividing weight in kilograms by squared height in metres and was used to derive BMI z-

scores using UK 1990 growth centiles[29]. Weight status was represented as a binary variable (normal/under-weight, overweight or obese), based on age- and sex-specific criteria provided by the International Obesity Task Force[30].

Potential confounding variables

Based on previous literature[31], potential confounding variables were considered from self-report data when adolescents were age 14 years. Ethnic group (white, mixed, Indian, Pakistani and Bangladeshi, Black/Black British, and other ethnic group), which was reclassified as (i) white and (ii) other. Parents reported their weekly household income (Organisation for Economic Co-operation and Development [OECD] equivalized income quintiles), highest maternal academic qualification level (degree plus, diploma, A levels, General Certificate of Secondary Education [GCSE] grade A to C, GCSE D to G, overseas only, and none), which was reclassified as (i) low, (ii) high, and (iii) Other qualifications, and adolescents' birthweight and gender during data collection at waves one and two (MCS1–MCS2). Adolescents reported their pubertal status, including growth spurt, body hair, and skin changes for both boys and girls, and additional factors such as voice breaking and facial hair for boys, and breast development and menarche for girls, at the age of 14 (MCS6) using a self-administered rating scale for pubertal development[32]. The following puberty categories were derived: prepubertal, early pubertal, midpubertal, late pubertal and postpubertal, which were dichotomised into (i) pre/early puberty, including 'prepubertal,' 'early pubertal,' and 'midpubertal,' and (ii) post/late pubertal, including 'late pubertal' and 'postpubertal' for the current analyses.

Ethics

Ethical approval has been granted for all MCS surveys via the National Health Service Research Ethics Committee system[33]. This ensure that all data is collected in line with ethical guidelines, including informed consent, confidentiality, and participant welfare. Written consent for data collection was obtained from parents during the early waves when cohort members were under the age of 16, and as participants grew older, their own consent was required for continued involvement[33]. Also, ethical issues extend to secondary data analysis, where researchers must adhere to guidelines for the appropriate and responsible use of data [33]. The MCS data can be obtained from the UK Data Service. After user and project registration with the UK Data Service, access to the MCS data was approved, and the datasets from sweeps 6 and 7 were downloaded onto an encrypted personal computer in anonymised STATA file format on November 12, 2020. Since the downloaded data were publicly available and fully anonymised, containing no personally identifiable information, no additional ethical approval was required for the current study[34].

Statistical analyses

Data management and analyses were conducted using Stata statistical software, version 18 (Stata, College Station, TX). Only participants with complete data for all the variables of interest at age 14 and 17 years were considered eligible for these analyses. Descriptive analysis was performed to compare demographic characteristics between the included and excluded populations using appropriate statistical methods (S1 Table). Pearson’s Chi-square test and independent samples t-tests were used to examine differences in baseline characteristics for

categorical and continuous variables, respectively. A p-value <0.05 was considered statistically significant.

Joint Correspondence Analysis (JCA) was used to explore patterns in categorical variables, involving four exposure measures (physical activity, screen-time, dietary behaviours, and sleep) with eight variables (MVPA, TV viewing, video game playing, social networking, sleep duration, and consumption of fruits, vegetables, and SSB). More details on the analyses used have been described elsewhere[35]. In brief, JCA was first conducted to reduce data dimensionality and to examine patterns and relationships in categorical variables. Then, K-means cluster analysis was utilised to create clusters of behaviours and was conducted using the coordinates obtained from the JCA. The algorithm was run with a predetermined number of clusters, k= 3, based on theoretical considerations and inspection of within-cluster sum of squares from the JCA. The resulting k-means clustering solution produced 3 distinct nonoverlapping clusters to group similar observations while maximising differences. The final number of clusters was determined and cross checked based on both JCA and k-means and their interpretability and distinctiveness of the cluster solution. The clusters' homogeneity was determined based on the sum of squared distances. Chi-square tests examined associations with sociodemographic variables.

Linear and logistic regression models, stratified by gender, were used to analyse the longitudinal associations between identified clusters at age 14 (baseline) and (1) BMI z-scores, (2) weight status categories, and (3) percent body fat at age 17 years, adjusted for maternal education, ethnicity, weekly family income, birthweight, puberty, baseline BMI z-score, baseline weight status, and baseline percent body fat, respectively.

Results

Participant characteristics

The participants excluded from the analysis differed in terms of ethnicity and socioeconomic status compared to those included (S1 Table). Complete data were available for 3,065 participants (52.5% girls), representing 16% of the original sample, as indicated in S1 Fig. The descriptive characteristics of the sample at age 14 are presented in Table 1. The majority of boys (84.8%) and girls (84.6%) were white British, just over half of participants (53.8%) had mothers with a higher level of education. There were no significant differences in ethnicity, OECD weekly family income, and maternal education between boys and girls.

Table 1: Participant characteristics and outcomes at ages 14 and 17 years

	**Girls** N= 1,608 (52.46%)	**Boys** N= 1,457 (47.54%)	**Total** (N= 3,065)	***P* value**
**Demographic characteristics**				
**Ethnicity**				
White	1,361 (84.64%)	1,235 (84.76%)	2,596 (84.70%)	0.924
Other	247 (15.36%)	222 (15.24%)	469 (15.30%)	
**OECD weekly family income**	446.37 (176.62)	457.27 (170.45)	451.55 (173.77)	0.0830
**NVQ Highest Level (all sweeps)**				
Low	675 (41.98%)	580 (39.81%)	1,255 (40.95%)	0.066
High	838 (52.11%)	811 (55.66%)	1,649 (53.80%)	
Other qualifications	95 (5.91%)	66 (4.53%)	161 (5.25%)	
**Birthweight**	3.32 (0.56)	3.45 (0.58)	3.38 (0.57)	< 0.001
**Puberty**				
Pre/early puberty	167 (10.43%)	986 (67.86%)	1,153 (37.75%)	< 0.001
Post/late pubertal	1,434 (89.57%)	467 (32.14%)	1,901 (62.25%)	
**Physical activity, sedentary behaviours, and sleep at age 14 years**				
**MVPA (mins/day)**	46.17 (29.88 – 70.29)	59.96 (36.92 – 95.00)	52.25 (32.31 – 81.48)	< 0.001
Total accelerometer wear time (both weekday and weekend)	47 (42.5 - 48)	46.25 (41.5 - 48)	47 (42 - 48)	0.0050

**Screen time (mins/weekday)**				
Watching TV / videos / computer	150 (90 - 240)	150 (90 - 240)	150 (90 - 240)	0.0039
Playing electronic games	15 (0 - 90)	150 (90 - 240)	90 (15 - 150)	< 0.001
Social networking sites	150 (45 - 240)	90 (15 - 150)	90 (45 - 240)	< 0.001
**Sleep duration (hrs /day)**				
< 9	705 (43.84%)	638 (43.79%)	1,343 (43.82%)	0.976
> 9	903 (56.16%)	819 (56.21%)	1,722 (56.18%)	
**Dietary intake**				
**Sweetened beverages (frequency/week)**				
< 3	1,049 (65.24%)	828 (56.83%)	1,877 (61.24%)	< 0.001
> 3	559 (34.76%)	629 (43.17%)	1,188 (38.76%)	
**At least 2 portions of fruit per day**				
Never / some days	1,070 (66.54%)	962 (66.03%)	2,032 (66.30%)	0.763
Every day	538 (33.46%)	495 (33.97%)	1,033 (33.70%)	
**At least 2 portions of vegetables per day**				
Never / some days	934 (58.08%)	879 (60.33%)	1,813 (59.15%)	0.207
Every day	674 (41.92%)	578 (39.67%)	1,252 (40.85%)	
**Outcomes**				
**Body fat % at 14**	26.70 (6.77)	16.27 (7.62)	21.74 (8.87)	< 0.001
**Body fat % at 17**	27.93 (8.08)	15.60 (7.93)	22.07 (10.09)	< 0.001
**BMI z-score at 14**	0.64 (1.14)	0.55 (1.15)	0.60 (1.15)	0.0202
**BMI z-score at 17**	1.12 (1.08)	1.21 (1.05)	1.16 (1.07)	0.0147
**BMI category 14**				
Normal/Under-weight	1,217 (75.68%)	1,138 (78.11%)	2,355 (76.84%)	0.272
Overweight	288 (17.91%)	238 (16.33%)	526 (17.16%)	
Obese	103 (6.41%)	81 (5.56%)	184 (6.00%)	
**BMI category 17**				
Normal/Under-weight	1,129 (70.21%)	1,090 (74.81%)	2,219 (72.40%)	0.017

Overweight	316 (19.65%)	244 (16.75%)	560 (18.27%)	
Obese	163 (10.14%)	123 (8.44%)	286 (9.33%)	

Abbreviations: OECD, organisation for economic co-operation and development; NVQ, national vocational qualification; MVPA, moderate to vigorous physical activity; mins, minutes; hrs, hours; BMI, body mass index.

JCA and K-means Cluster Analysis

The JCA results revealed two dimensions that explained 92.7% of the total variance within the dataset (i.e. the extent to which the two dimensions explain the variability among the variables included in the analysis). The scatter plot (Fig 1) displays the positions of the observations and the relationships between the physical activity, dietary, screen-time and sleep variables. K-means clustering solution produced three distinct clusters (Fig 2), which were termed healthy cluster, mixed cluster, and unhealthy cluster.

Fig 1: Joint correspondence analysis plot of health behaviours variables.

Fig 2: K-means cluster analysis at age 14.

The characteristics of each of the three clusters are described in Table 2. For both boys and girls, Cluster 1 (healthy cluster) was characterised by a higher proportion of adolescents who met the physical activity guidelines, watched screens for less than 2 hours per day, had a sleep duration of more than 9 hours, consumed SSB less than 3 times a week, and consumed 2 portions of fruits and vegetables daily. Cluster 2 (mixed cluster) was characterised by values of health behaviours and health outcomes that fall between those observed in the healthy and unhealthy clusters. Cluster 3 (unhealthy cluster) was characterised by a higher proportion of adolescents who did not met the physical activity guidelines, never/some days ate 2 portions of fruits and vegetables, consumed SSB more than 3 times a week, watched screens for more than 2 hours, and had sleep durations of less than 9 hours.

At age 14, a higher proportion of boys were in the unhealthy cluster (53.0%) compared to girls, and a higher proportion of girls in the healthy cluster (55.9%) compared to boys. For both boys and girls, a higher proportion of adolescents with mothers who had higher educational levels were in the healthy clusters compared to those whose mothers had lower or ‘other’ levels of education (69.6% for boys, and 65.4% for girls). A higher proportion of adolescents with mothers with lower educational levels were in the unhealthy cluster compared to those whose mothers who had higher or ‘other’ levels of education (48.9% for boys, and 50.1% for girls).

Table 2: Demographic and behavioural characteristics of behavioural clusters at age 14 years split by gender

**Cluster**	**Cluster 1 Healthy Cluster** N= 925 (30.18%)	**Cluster 2 Mixed Cluster** N= 1346 (43.92%)	**Cluster 3 Unhealthy Cluster** N= 794 (25.91%)	***P* value**
**Gender**				0.001
Boys	408 (44.11%)	628 (46.66%)	421 (53.02%)	
Girls	517 (55.89%)	718 (53.34%)	373 (46.98%)	
**Gender** Boys				
**Ethnicity**				0.048
White	361 (88.48%)	523 (83.28%)	351 (83.37%)	
Other	47 (11.52%)	105 (16.72%)	70 (16.63%)	
**Puberty**				0.441
Pre/early puberty	285 (70.02%)	425 (67.78%)	276 (65.87%)	
Post/late pubertal	122 (29.98%)	202 (32.22%)	143 (34.13%)	
**OECD weekly family income**	524 (156)	450 (172)	403 (161)	<0.001
**NVQ Highest Level (all sweeps)**				<0.001
Low	117 (28.68%)	257 (40.92%)	206 (48.93%)	
High	284 (69.61%)	341 (54.30%)	186 (44.18%)	

Other qualifications	7 (1.72%)	30 (4.78%)	29 (6.89%)	
**Physical activity**				<0.001
Not meeting the guidelines*	155 (37.99%)	318 (50.64%)	256 (60.81%)	
Meeting the guidelines*	253 (62.01%)	310 (49.36%)	165 (39.19%)	
**Screen time (hrs/weekday)**				
**Watching TV / videos / computer**				<0.001
< 2	258 (63.24%)	204 (32.48%)	68 (16.15%)	
> 2	150 (36.76%)	424 (67.52%)	353 (83.85%)	
**Playing electronic games**				<0.001
< 2	301 (73.77%)	224 (35.67%)	54 (12.83%)	
> 2	107 (26.23%)	404 (64.33%)	367 (87.17%)	
**Social networking sites**				<0.001
< 2	357 (87.50%)	444 (70.70%)	170 (40.38%)	
> 2	51 (12.50%)	184 (29.30%)	251 (59.62%)	
**Sleep duration (hrs/day)**				<0.001
< 9	130 (31.86%)	264 (42.04%)	244 (57.96%)	
> 9	278 (68.14%)	364 (57.96%)	177 (42.04%)	
**Dietary intake**				
**Sweetened beverages (frequency/week)**				<0.001
< 3	342 (83.82%)	398 (63.38%)	88 (20.90%)	
> 3	66 (16.18%)	230 (36.62%)	333 (79.10%)	
**At least 2 portions of fruit per day**				<0.001
Never / some days	100 (24.51%)	455 (72.45%)	407 (96.67%)	
Every day	308 (75.49%)	173 (27.55%)	14 (3.33%)	
**At least 2 portions of vegetables per day**				<0.001
Never / some days	64 (15.69%)	420 (66.88%)	395 (93.82%)	
Every day	344 (84.31%)	208 (33.12%)	26 (6.18%)	
**Indicators of adiposity at age 14**				

Body fat % at 14	14.91 (6.13)	16.70 (8.26)	16.95 (7.79)	<0.001
BMI z-score at 14	0.42 (1.06)	0.57 (1.20)	0.64 (1.17)	0.0002
BMI category 14				0.032
Normal/Under-weight	336 (82.35%)	483 (76.91%)	319 (75.77%)	
Overweight	61 (14.95%)	104 (16.56%)	73 (17.34%)	
Obese	11 (2.70%)	41 (6.53%)	29 (6.89%)	
**Indicators of adiposity at age 17**				
Body fat % at 17	14.66 (6.72)	15.84 (8.59)	16.17 (7.91)	<0.001
BMI z-score at 17	1.16 (0.92)	1.21 (1.12)	1.26 (1.08)	0.0027
BMI category 17				0.001
Normal/Under-weight	322 (78.92%)	466 (74.20%)	302 (71.73%)	
Overweight	71 (17.40%)	98 (15.61%)	75 (17.81%)	
Obese	15 (3.68%)	64 (10.19%)	44 (10.45%)	
**Gender** Girls				
	**Cluster 1 Healthy Cluster**	**Cluster 2 Mixed Cluster**	**Cluster 3 Unhealthy Cluster**	***P* value**
**Ethnicity**				0.091
White	452 (87.43%)	601 (83.70%)	308 (82.57%)	
Other	65 (12.57%)	117 (16.30%)	65 (17.43%)	
**Puberty**				<0.001
Pre/early puberty	76 (14.76%)	57 (7.98%)	34 (9.14%)	
Post/late pubertal	439 (85.24%)	657 (92.02%)	338 (90.86%)	
**OECD weekly family income**	508 (173)	434 (171)	384 (167)	<0.001
**NVQ Highest Level (all sweeps)**				<0.001
Low	165 (31.91%)	323 (44.99%)	187 (50.13%)	
High	338 (65.38%)	351 (48.89%)	149 (39.95%)	
Other qualifications	14 (2.71%)	44 (6.13%)	37 (9.92%)	
**Physical activity**				<0.001

Not meeting the guidelines*	278 (53.77%)	490 (68.25%)	273 (73.19%)	
Meeting the guidelines*	239 (46.23%)	228 (31.75%)	100 (26.81%)	
**Screen time (hrs/weekday)**				
**Watching TV / videos / computer**				<0.001
< 2	274 (53.00%)	201 (27.99%)	41 (10.99%)	
> 2	243 (47.00%)	517 (72.01%)	332 (89.01%)	
**Playing electronic games**				<0.001
< 2	494 (95.55%)	604 (84.12%)	220 (58.98%)	
> 2	23 (4.45%)	114 (15.88%)	153 (41.02%)	
**Social networking sites**				<0.001
< 2	352 (68.09%)	279 (38.86%)	52 (13.94%)	
> 2	165 (31.91%)	439 (61.14%)	321 (86.06%)	
**Sleep duration (hrs/day)**				<0.001
< 9	185 (35.78%)	317 (44.15%)	203 (54.42%)	
> 9	332 (64.22%)	401 (55.85%)	170 (45.58%)	
**Dietary intake**				
**Sweetened beverages (frequency/week)**				<0.001
< 3	462 (89.36%)	496 (69.08%)	91 (24.40%)	
> 3	55 (10.64%)	222 (30.92%)	282 (75.60%)	
**At least 2 portions of fruit per day**				<0.001
Never / some days	114 (22.05%)	589 (82.03%)	367 (98.39%)	
Every day	403 (77.95%)	129 (17.97%)	6 (1.61%)	
**At least 2 portions of vegetables per day**				<0.001
Never / some days	65 (12.57%)	513 (71.45%)	356 (95.44%)	
Every day	452 (87.43%)	205 (28.55%)	17 (4.56%)	
**Indicators of adiposity at age 14**				
Body fat % at 14	26.08 (6.82)	26.52 (6.50)	27.91 (7.06)	<0.001
BMI z-score at 14	0.58 (1.12)	0.62 (1.11)	0.80 (1.20)	0.0002
BMI category 14				0.046
Normal/Under-weight	398	559	260	

	(76.98%)	(77.86%)	(69.71%)	
Overweight	88 (17.02%)	118 (16.43%)	82 (21.98%)	
Obese	31 (6.00%)	41 (5.71%)	31 (8.31%)	
**Indicators of adiposity at age 17**				
Body fat % at 17	27.47 (7.62)	27.69 (7.92)	29.03 (8.87)	<0.001
BMI z-score at 17	1.05 (1.04)	1.09 (1.07)	1.24 (1.16)	0.0027
BMI category 17				0.017
Normal/Under-weight	375 (72.53%)	518 (72.14%)	236 (63.27%)	
Overweight	98 (18.96%)	129 (17.97%)	89 (23.86%)	
Obese	44 (8.51%)	71 (9.89%)	48 (12.87%)	

Abbreviations: OECD, organisation for economic co-operation and development; NVQ, national vocational qualification; hrs, hours; BMI, body mass index. *>60 minutes of moderate to vigorous physical activity per day.

Tables 3 (unadjusted models) and 4 (adjusted models) represent the longitudinal associations between clusters at age 14 and changes in BMI z-score, weight status, and percent body fat between ages 14 and 17. Change in BMI z-scores were significantly lower among boys in the mixed and unhealthy clusters compared to those in the healthy cluster (β = - 0.08; 95% CI = (-0.146, - 0.005)), β = - 0.08; 95% CI = (- 0.158, - 0.001)), respectively). No significant changes were observed for weight status and percent body fat for both sexes.

Table 3: Association between behaviour clusters at age 14 and adiposity markers at age 17, unadjusted models

	BMI z-score $^{\Delta}$		*P* value	Weight status $^{\Delta}$		*P* value	% BF $^{\Delta}$		*P* value
	β	95% CI		Odds ratio	95% CI		β	95% CI	
Boys									
Cluster 1 Healthy (Reference)									
Cluster 2 Mixed	0.051	(- 0.081) - (0.182)	0.448	1.302	0.967 - 1.753	0.083	1.177	0.190 - 2.163	**0.019**

Cluster 3 Unhealthy	0.103	(- 0.040) - (0.247)	0.159	1.475	1.073 - 2.029	**0.017**	1.512	0.434 - 2.590	**0.006**
Girls									
Cluster 1 Healthy (Reference)									
Cluster 2 Mixed	0.040	(- 0.083) - (0.162)	0.530	1.020	0.792 - 1.313	0.880	0.221	(- 0.691) - (0.133)	0.635
Cluster 3 Unhealthy	0.191	(0.046) - (0.335)	**0.010**	1.533	1.152 - 2.040	**0.003**	1.556	0.483 - 2.630	**0.005**

Abbreviations: BMI, body mass index; %BF, percent body fat; 95%CI: 95% confidence interval.

Table 4: Association between behaviour clusters at age 14 and adiposity markers at age 17, adjusted models

	BMI z-score $^{\Delta}$		*P* value	Weight status $^{\Delta}$		*P* value	% BF $^{\Delta}$		*P* value
	β	95% CI		Odds ratio	95% CI		β	95% CI	
Boys									
Cluster 1 Healthy (Reference)									
Cluster 2 Mixed	- 0.08	(- 0.146) – (-0.005)	**0.036**	0.96	0.644 - 1.428	0.835	- 0.33	(- 1.044) - (0.386)	0.367
Cluster 3 Unhealthy	- 0.08	(- 0.158) – (- 0.001)	**0.048**	1.08	0.700 - 1.671	0.725	- 0.31	(- 1.107) - (0.491)	0.450
Girls									
Cluster 1 Healthy (Reference)									
Cluster 2 Mixed	0.001	(- 0.063) - (0.065)	0.976	0.96	0.673 - 1.369	0.820	-0.289	(- 0.874) - (0.296)	0.333
Cluster 3 Unhealthy	-0.007	(- 0.083) - (0.069)	0.853	1.20	0.792 -1.810	0.393	-0.287	(- 0.988) - (0.414)	0.422

Abbreviations: BMI, body mass index; %BF, percent body fat; 95%CI: 95% confidence interval. Model was adjusted for maternal education, weekly family income, ethnicity, birthweight and puberty, baseline BMI z-score, baseline weight status, baseline percent body fat, respectively.

Discussion

This study identified and described the clustering of screen time, physical activity, dietary behaviours and sleep among adolescents and examined the associations with 3-year changes in indicators of adiposity. This study presents a novel approach by examining adolescence, a critical period of life, and exploring the associations between clusters of health behaviours and changes in weight over a 3-year period in a UK sample, with the inclusion of sleep patterns within the analysis of behaviour clustering. The present study identified three clusters, a 'healthy cluster,' 'mixed cluster,' and 'unhealthy cluster,' with the unhealthy cluster being the most prevalent among boys and the healthy cluster being the most prevalent among girls. We found that boys in the mixed and unhealthy clusters at age 14 years had a significantly lower change in BMI z-score compared to those in the healthy cluster. No other associations were identified between clusters of health behaviours and changes in indicators of adiposity.

The present study found that the prevalence of adolescents in the healthy clusters at 14 years of age was around 30%, while the prevalence of adolescents in the unhealthy clusters was just below 30%[11,15]. In support of this finding, previous systematic reviews show that the majority of adolescents are categorised into mixed clusters, where one or more healthy behaviour(s) coexists with one or more unhealthy behaviour(s), highlighting the intricate nature of adolescents' health behaviours. The present study also revealed that adolescents of mothers with higher education were more likely found in the healthy cluster and adolescents of mothers

with lower education were more likely found in the unhealthy cluster; an observation also consistently observed in previous research[11,15,36]. In contrast to a recent review[15], we observed a higher prevalence of girls in the healthy cluster, while boys were predominantly in the unhealthy cluster. This maybe attributed, in part, to variations in the methods used to assess behaviours (e.g. self-report vs. device measure). For example, self-reported PA measures in previous studies indicated a higher occurrence of unfavourable health behaviours among girls compared to boys[37,38]. Further prospective research conducted with more objective measures such as those used in the study are needed to provide a comprehensive understanding of the factors influencing different demographic groups.

A limited number of previous studies indicate a longitudinal association between persistent engagement in unhealthy behaviours and obesity-related measures[39–41]. For example, a study conducted in Germany explored the longitudinal association between patterns of self-reported health-related behaviours and changes in weight status and self-rated health among adolescents. It revealed that adolescents belonging to a poor health-related behaviour pattern characterised by low activity levels, high media use, and poor diet quality experienced the most significant increase in overweight prevalence over a 6-year period. While the immediate impact of clusters of health behaviours on change in obesity related measures between 14 and 17 may not be apparent in the present study, the prevalence of these unhealthy behaviours underscores the need for proactive intervention and preventive measures to mitigate their potential long-term impact on health outcomes.[15] Addressing these behaviours early on is crucial to fostering healthier lifestyles and averting adverse health consequences in the future.

The present study revealed an unexpected trend where adolescent boys in the mixed and unhealthy clusters at baseline had a smaller change in BMI z-score between ages 14-17 compared to those in the healthy cluster. Although this finding diverges from conventional expectations, we posit that it is partially explained by boys with unhealthy behaviours having a higher BMI z-score (0.64 (SD = 1.17) at age 14 compared with the other cluster (healthy (0.42 (SD = 1.06)) and mixed (0.57 (SD = 1.20)), consequently experiencing less subsequent gain than their counterparts with healthier behaviours. Despite the slower gain in BMI z-scores in these clusters they still have higher mean BMI z-scores at age 17 than the other healthy cluster.

One crucial aspect that may require further emphasis is the substantial proportion of clusters categorised as overweight or obese at age 17. This observation sheds light on the significant prevalence of overweight and obesity within the studied population during late adolescence. Such a high prevalence underscores the pressing need for targeted interventions and preventive measures aimed at addressing obesity-related issues among adolescents. Understanding the factors within these clusters contributing to the persistence or development of overweight and obesity in this age group is important for effective public health strategies and interventions to promote healthy weight management and mitigate associated health risks.

A second hypothesis to explain the finding that adolescent boys in the mixed and unhealthy clusters at baseline had less change in BMI z-score longitudinally, may be attributed to the 'regression to the mean' (RTM) phenomenon[42]. The principle of RTM predicts greater mean weight change will occur in those participants with more extreme baseline body weight, leading to an inverse association between baseline body weight and expected weight change even without any intervention. This RTM confounds the interpretation of weight change,

especially in those subjects whose baseline weight differs most from the population mean. In our study, this suggests that the initially lower prevalence of obesity in the 'healthy' cluster may have represented an outlier. Over time, the BMI values are converging towards the average, contributing to the unexpected longitudinal BMI change in this cluster. It's important to note the absence of this pattern when using the objective percent body fat measure, highlighting the challenges associated with using BMI for assessing obesity in adolescents due to its limitations in accurately capturing variations in body composition and the potential for misclassifying young people's weight status.[43]

These findings can only be partially compared to a few studies that explored longitudinal associations between health behaviour clusters and adiposity change over time due to differences in the variables included across studies[39–41]. Notably, in contrast to the present study, a recent longitudinal study of 1634 Spanish adolescents aged 8-18 years found that participants belonging to the healthy behaviour cluster, characterised by low screen and sedentary time and high MVPA and adherence to the Mediterranean diet, showed significantly lower percent body fat two years later compared with the other profiles[16]. Another study of 1642 German adolescents showed that health behaviour clusters of low activity level with high media use and low diet quality had the strongest increase in prevalence of overweight over a period of six years[41]. Similarly, Gubbels et al also revealed a longitudinal positive association between a ‘sedentary (TV viewing)/snacking’ pattern at five years of age and BMI Z-score at 7–8 years[40]. The inconsistency in the evidence with the present study can be partly linked to the utilisation of different methods for assessing health behaviours[44], as well as the different age group focuses of the studies, and different time frames of follow up. Moreover, many large-scale population-based studies rely on respondent self-reports to measure physical activity, which are susceptible to both recall and social desirability bias[45]. Also, as the

questions assessing SSB consumption do not measure portion size, the under-reporting of SSB intake with the over-reporting of fruits and vegetables by adolescents with a higher BMI might have occurred, because overweight/obese adolescents are inclined to under-report their unhealthy dietary intake more often than adolescents with normal weight[46].

Strengths and limitations

This present study has several methodological strengths. First, it is based on data from a large, representative cohort of adolescents with a three year follow up. Second, the longitudinal study design and adjustment for baseline adiposity lowers the risk of reverse causation[47]. Third, percent body fat, via calibrated Tanita BF-522W scales, was utilised which is a more direct measure of adiposity compared to BMI[48]. Lastly, device-based measures of MVPA reduced the bias associated with self-report[49]. It is important to consider the following limitations when interpreting the results. We acknowledge that a substantial number of participants were excluded from the analysis due to missing accelerometer data, consistent with previous research using this methodology[50]. The participants excluded from the analysis differed in terms of ethnicity and socioeconomic status compared to those included. Moreover, relying on just two valid days of accelerometer data, including one weekend day and one weekday, to measure MVPA may not adequately reflect an individual's regular physical activity patterns[51]. However, it is worth noting that some existing data do suggest that a measurement period of two days can provide reliable insights into overall physical activity levels[52]. Additionally, screen time habits in young adolescents may have changed since they were initially measured in 2015 and 2016 in the MCS[31]. Also, relying on fruits, vegetables and SSB to measure dietary behaviours and excluding other important diet components is a limitation in relation to understanding overall energy intakes and diversity of

diet. It is important also to emphasise that even young people within the healthy cluster did not necessarily adhere to recommended dietary guidelines (i.e. 5 portions of fruit and vegetables daily). There could also have been a measurement error with the dietary behaviours data because the self-reported questionnaire used are prone to biases, such as attention and recall bias[46]. Furthermore, the questions evaluating SSB consumption do not account for portion size, which could contribute to the under-reporting of SSB intake. Finally, we are not able to examine whether clusters tracked between the ages of 14 and 17 due to limitations with the available MCS data.

Conclusion

To our knowledge, this is the first longitudinal study among older adolescents in the UK to examine the associations between clusters of health behaviours and change in indicators of adiposity over time. Adolescents in the healthy cluster had lower BMI z-scores and percent body fat at age 14 years than those in the unhealthy cluster, and they maintained this lower level at age 17. Our findings show that for most of the indicators of adiposity measured across both boys and girls that there were no significant differences in change in these outcomes between the health behaviour clusters between 14-17 years. This was with the exception of BMI z-score where there was a marginally statistically significant finding with a small effect size showing the unhealthy and mixed clusters to have had less change in BMI z-scores during this period from a relatively higher start in BMI z-scores compared to the healthy cluster. To support future research, there is a clear need to establish prospective research conducted with better objective measures of diet, sleep, and screen time. Also, as adolescents' health behaviours are complex and undergo changes with age[53], further research is necessary to

comprehensively understand and analyse these evolving patterns. Additionally, examining the stability of cluster membership over time is needed.

Acknowledgements

Not applicable.

References

1. Adela Hruby, PhD M, Frank B. Hu, MD, PhD M. The Epidemiology of Obesity: A Big Picture. Pharmacoeconomics. 2015;33(7):673–89.
2. NHS Digital. Statistics on Obesity, Physical Activity and Diet, England, 2020 [Internet]. NHS Digital. 2020 [cited 2023]. Available from: https://digital.nhs.uk
3. Pearson N, Sherar LB, Hamer M. Prevalence and Correlates of Meeting Sleep, Screen-Time, and Physical Activity Guidelines among Adolescents in the United Kingdom. JAMA Pediatr. 2019;173(10):993–4.
4. Frech A. Healthy behavior trajectories between adolescence and young adulthood. Adv Life Course Res. 2012;17(2):59–68.
5. Elsenburg LK, Corpeleijn E, van Sluijs EMF, Atkin AJ. Clustering and Correlates of Multiple Health Behaviours in 9–10 Year Old Children. Broyles ST, editor. PLoS One. 2014;9(6):e99498.
6. Pereira S, Katzmarzyk PT, Gomes TN, Borges A, Santos D, Souza M, et al. Profiling physical activity, diet, screen and sleep habits in Portuguese children. Nutrients. 2015;7(6):4345–62.
7. Saunders TJ, Gray CE, Poitras VJ, Chaput JP, Janssen I, Katzmarzyk PT, et al. Combinations of physical activity, sedentary behaviour and sleep: Relationships with health indicators in school-aged children and youth. Vol. 41, Applied Physiology, Nutrition and Metabolism. Canadian Science Publishing; 2016. p. S283–93.
8. Ottevaere C, Huybrechts I, Benser J, De Bourdeaudhuij I, Cuenca-Garcia M, Dallongeville J, et al. Clustering patterns of physical activity, sedentary and dietary behavior among European adolescents: The HELENA study. BMC Public Health. 2011;11:328.
9. Mayne SL, Virudachalam S, Fiks AG. Clustering of unhealthy behaviors in a nationally representative sample of U.S. children and adolescents. Prev Med (Baltim). 2020;130.
10. Ottevaere C, Huybrechts I, Benser J, De Bourdeaudhuij I, Cuenca-Garcia M, Dallongeville J, et al. Clustering patterns of physical activity, sedentary and dietary behavior among European adolescents: The HELENA study. BMC Public Health. 2011;11:328.
11. Leech RM, Mcnaughton SA, Timperio A. The clustering of diet, physical activity and sedentary behavior in children and adolescents: a review. International journal of behavioral nutrition and physical activity. 2014;11(1):1–9.
12. Leech RM, McNaughton SA, Timperio A. Clustering of children's obesity-related behaviours: Associations with sociodemographic indicators. Eur J Clin Nutr. 2014;68(5):623–8.
13. Dumuid D, Olds T, Lewis LK, Martin-Fernández JA, Barreira T, Broyles S, et al. The adiposity of children is associated with their lifestyle behaviours: a cluster analysis of school-aged children from 12 nations. Pediatr Obes. 2018;13(2):111–9.
14. Moreira NF, da Veiga GV, Santaliestra-Pasías AM, Androutsos O, Cuenca-García M, de Oliveira ASD, et al. Clustering of multiple energy balance related behaviors is associated with body fat composition indicators in adolescents: Results from the HELENA and ELANA studies. Appetite. 2018;120:505–13.

15. Alosaimi N, Sherar LB, Griffiths P, Pearson N. Clustering of diet, physical activity and sedentary behaviour and related physical and mental health outcomes: a systematic review. BMC Public Health. 2023;23(1):1572.
16. Sánchez-Oliva D, Grao-Cruces A, Carbonell-Baeza A, Cabanas-Sánchez V, Veiga OL, Castro-Piñero J. Lifestyle Clusters in School-Aged Youth and Longitudinal Associations with Fatness: The UP&DOWN Study. Journal of Pediatrics. 2018;203:317-324.e1.
17. Pérez-Rodrigo C, Gil Á, González-Gross M, Ortega R, Serra-Majem L, Varela-Moreiras G, et al. Clustering of Dietary Patterns, Lifestyles, and Overweight among Spanish Children and Adolescents in the ANIBES Study. Nutrients [Internet]. 2015;8(1):11. Available from: www.mdpi.com
18. Leech RM, McNaughton SA, Timperio A. The clustering of diet, physical activity and sedentary behavior in children and adolescents: A review. International Journal of Behavioral Nutrition and Physical Activity. 2014;11(1):1–9.
19. Santaliestra-Pasías AM, Mouratidou T, Reisch L, Pigeot I, Ahrens W, Mårild S, et al. Clustering of lifestyle behaviours and relation to body composition in European children. The IDEFICS study. Eur J Clin Nutr [Internet]. 2015;69(7):811–6. Available from: www.nature.com
20. Pearson N, Griffiths P, Biddle SJ, Johnston JP, McGeorge S, Haycraft E. Clustering and correlates of screen-time and eating behaviours among young adolescents. BMC Public Health. 2017;17(1):533.
21. Pearson N, Biddle SJH, Griffiths P, Johnston JP, Haycraft E. Clustering and correlates of screen-time and eating behaviours among young children. BMC Public Health [Internet]. 2018;18(1):753. Available from: https://bmcpublichealth.biomedcentral.com/articles/10.1186
22. Connelly R, Platt L. Cohort profile: UK Millennium Cohort Study (MCS). Int J Epidemiol. 2014;43(6):1719–25.
23. Heywood J. Millennium Cohort Study Physical Activity: Accelerometer dataset MCS6(2015) USER GUIDE [Internet]. 2018. Available from: www.cls.ioe.ac.uk
24. van Hees VT, Gorzelniak L, Dean León EC, Eder M, Pias M, Taherian S, et al. Separating Movement and Gravity Components in an Acceleration Signal and Implications for the Assessment of Human Daily Physical Activity. PLoS One. 2013;8(4).
25. da Silva IC, van Hees VT, Ramires V V, Knuth AG, Bielemann RM, Ekelund U, et al. Physical activity levels in three Brazilian birth cohorts as assessed with raw triaxial wrist accelerometry. Int J Epidemiol. 2014;43(6):1959–68.
26. Piercy KL, Troiano RP, Ballard RM, Carlson SA, Fulton JE, Galuska DA, et al. The Physical Activity Guidelines for Americans. JAMA [Internet]. 2018;320(19):2020. Available from: http://jama.jamanetwork.com/article.aspx?doi=10.1001
27. Strasburger VC, Mulligan DA, Altmann TR, Brown A, Christakis DA, Clarke-Pearson K, et al. Policy statement - Children, adolescents, obesity, and the media. Vol. 128, Pediatrics. American Academy of Pediatrics; 2011. p. 201–8.
28. Roesler A, Rojas N, Falbe J. Sugar-Sweetened Beverage Consumption, Perceptions, and Disparities in Children and Adolescents. J Nutr Educ Behav. 2021;53(7):553–63.
29. Cole TJ, Freeman J V, Preece MA, Dunn M. Body mass index reference curves for the UK, 1990. Vol. 73, Archives of Disease in Childhood. 1995.
30. Cole TJ, Lobstein T. Extended international (IOTF) body mass index cut-offs for thinness, overweight and obesity. Pediatr Obes. 2012;7(4):284–94.
31. Fitzsimons E. Millennium Cohort Study Sixth Survey 2015-2016 User Guide (First Edition). 2017.

32. Carskadon MA, Acebo C. A self-administered rating scale for pubertal development. Journal of Adolescent Health. 1993;14(3):190–5.
33. Hansen K, Calderwood L, Mostafa T, Platt L, Rosenberg R, Smith K. Millennium Cohort Study: A guide to the datasets (8th Ed.) [Internet]. London; 2014. Available from: www.cls.ioe.ac.uk
34. Dale A, Wathan J, Higgins V. Secondary Analysis of Quantitative Data Sources. In: The SAGE Handbook of Social Research Methods. 1 Oliver's Yard, 55 City Road, London EC1Y 1SP United Kingdom : SAGE Publications Ltd; 2008. p. 520–35.
35. Greenacre M. FROM CORRESPONDENCE ANALYSIS TO MULTIPLE AND JOINT CORRESPONDENCE ANALYSIS. Available at SSRN 847664; 2005 Sep.
36. D'Souza NJ, Kuswara K, Zheng M, Leech R, Downing KL, Lioret S, et al. A systematic review of lifestyle patterns and their association with adiposity in children aged 5–12 years. Obesity Reviews. 2020;21(8):e13029.
37. de Mello GT, Silva KS, da Costa RM, de Sousa ACFC, Benedet J, Del Duca GF, et al. How physical activity, diet, and sedentary behavior cluster according to age in adolescents? Sport Sci Health. 2022;18(2):499–507.
38. Cureau F V., Sparrenberger K, Bloch K V., Ekelund U, Schaan BD. Associations of multiple unhealthy lifestyle behaviors with overweight/obesity and abdominal obesity among Brazilian adolescents: A country-wide survey. Nutrition, Metabolism and Cardiovascular Diseases. 2018;28(7):765–74.
39. Leech RM, McNaughton SA, Timperio A. Clustering of diet, physical activity and sedentary behaviour among Australian children: Cross-sectional and longitudinal associations with overweight and obesity. Int J Obes. 2015;39(7):1079–85.
40. Gubbels JS, Kremers SPJ, Goldbohm RA, Stafleu A, Thijs C. Energy balance-related behavioural patterns in 5-year-old children and the longitudinal association with weight status development in early childhood. Public Health Nutr. 2012;15(8):1402–10.
41. Spengler S, Mess F, Schmocker E, Woll A. Longitudinal associations of health-related behavior patterns in adolescence with change of weight status and self-rated health over a period of 6 years: Results of the MoMo longitudinal study. BMC Pediatr. 2014;14:242.
42. Thomas DM, Clark N, Turner D, Siu C, Halliday TM, Hannon BA, et al. Best (but oft-forgotten) practices: identifying and accounting for regression to the mean in nutrition and obesity research. Am J Clin Nutr. 2020;111(2):256–65.
43. Sathiarajan D, Narayana Murthy MR, Gopi A, . A, Y. S. K. Body mass index and body fat percentage in determining overweight and obesity among school going adolescents of urban Mysuru, Karnataka: a cross-sectional study. Int J Community Med Public Health. 2023;10(2):734–9.
44. Dall PM, Coulter EH, Fitzsimons CF, Skelton DA, Chastin S. TAxonomy of Self-reported Sedentary behaviour Tools (TASST) framework for development, comparison and evaluation of self-report tools: content analysis and systematic review on behalf of the Seniors USP Team. BMJ Open [Internet]. 2017;7(4). Available from: http://dx.doi.org/10.1136
45. Hidding LM, Altenburg TM, Mokkink LB, Terwee CB, Chinapaw MJM. Systematic Review of Childhood Sedentary Behavior Questionnaires: What do We Know and What is Next? Sports Medicine. 2017;47(4):677–99.
46. Garaulet M, Martínez A, Victoria F, Pérez–Llamas F, Ortega RM, Zamora S. Differences in Dietary Intake and Activity Level Between Normal-Weight and Overweight or Obese Adolescents. J Pediatr Gastroenterol Nutr. 2000;30(3):253–8.

47. Zapf D, Dormann C, Frese M. Longitudinal studies in organizational stress research: A review of the literature with reference to methodological issues. J Occup Health Psychol. 1996;1(2):145–69.
48. Harbin G, Shenoy C, Olson J. Ten-year comparison of BMI, body fat, and fitness in the workplace. Am J Ind Med. 2006;49(4):223–30.
49. Lines RLJ, Ntoumanis N, Thøgersen-Ntoumani C, McVeigh JA, Ducker KJ, Fletcher D, et al. Cross-sectional and longitudinal comparisons of self-reported and device-assessed physical activity and sedentary behaviour. J Sci Med Sport. 2020;23(9):831–5.
50. Kontostoli E, Jones AP, Pearson N, Foley L, Biddle SJH, Atkin AJ. The Association of Contemporary Screen Behaviours with Physical Activity, Sedentary Behaviour and Sleep in Adolescents: a Cross-sectional Analysis of the Millennium Cohort Study. Int J Behav Med. 2023;30(1):122–32.
51. Cain KL, Sallis JF, Conway TL, Dyck D Van, Calhoon L. Using Accelerometers in Youth Physical Activity Studies: A Review of Methods. J Phys Act Health [Internet]. 2013;10(3):437–50. Available from: http://riskfactor.cancer.gov
52. Lee PH. Determining the Optimal Number of Wearing-Days Given a Fixed Number of Accelerometers in Population-Level Study. J Epidemiol. 2019;29(11):432–43.
53. Ames ME, Leadbeater BJ, MacDonald SWS. Health behavior changes in adolescence and young adulthood: Implications for cardiometabolic risk. Health Psychology. 2018;37(2):103–13.

Supporting information

S1 Table. Descriptive analysis of demographic characteristics comparing the included and excluded populations. Abbreviations: OECD, organisation for economic co-operation and development; NVQ, national vocational qualification.

S1 Fig. Sample selection flow-chart.

S2 Table. Demographic and behavioural characteristics of behavioural clusters at age 14 years. Abbreviations: OECD, organisation for economic co-operation and development; NVQ, national vocational qualification; hrs, hours; BMI, body mass index. *>60 minutes of moderate to vigorous physical activity per day.

Click here to access/download;Figure;Fig 1.docx

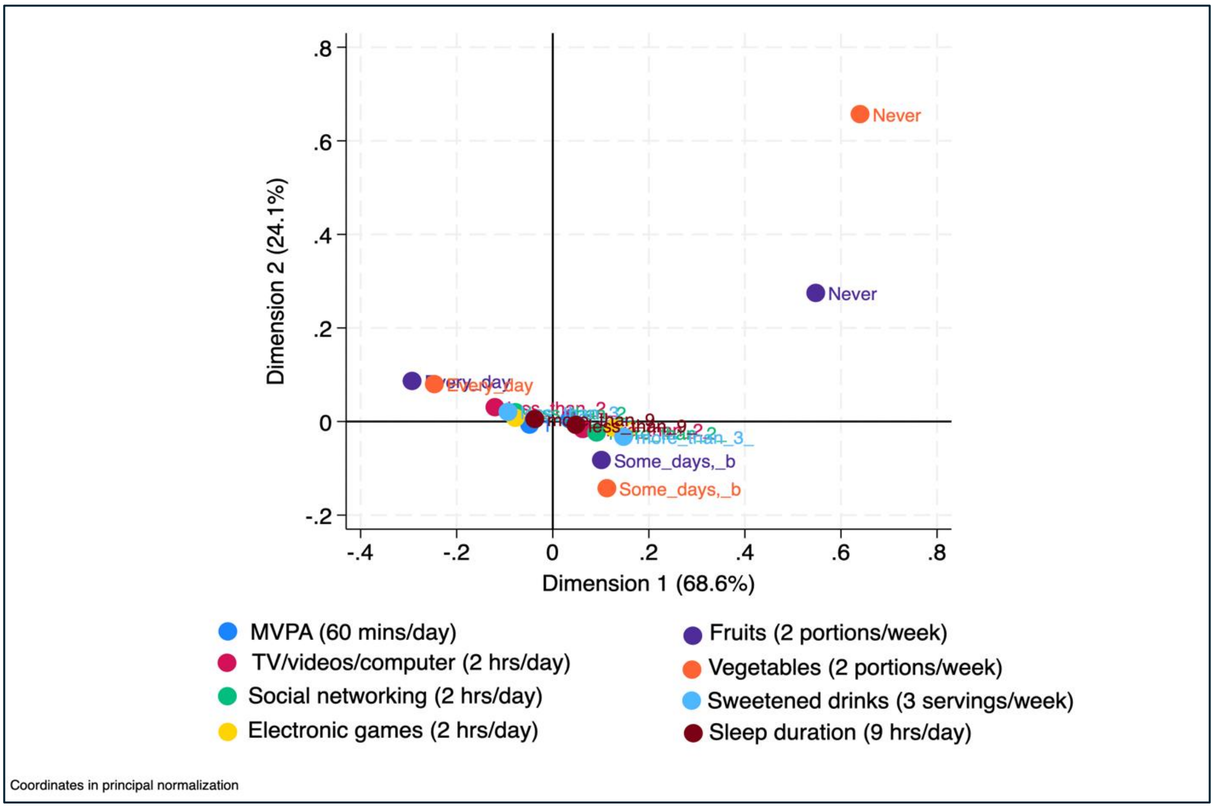

Fig 2

Click here to access/download;Figure;Fig 2.docx

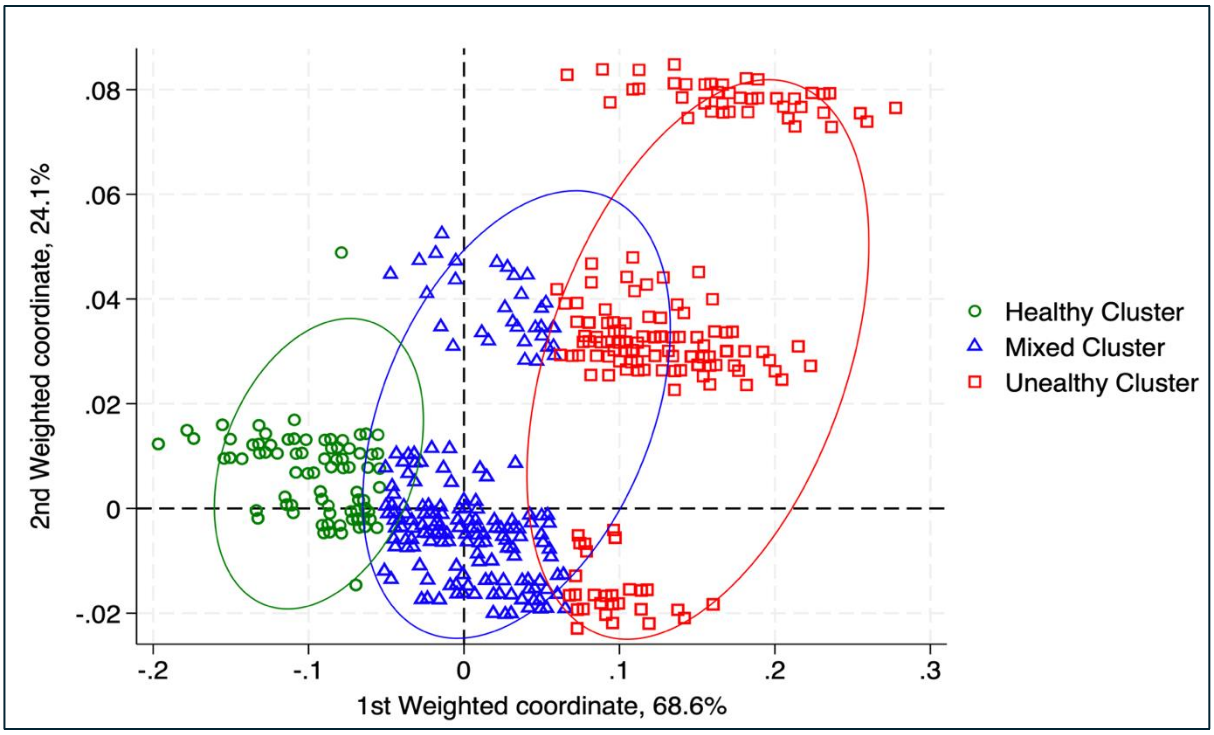

S2 Table

Click here to access/download
Supporting Information
S2_Table.docx

S1 Table

Click here to access/download
Supporting Information
S1_Table.docx

S1 Fig

Click here to access/download
Supporting Information
S1_Fig.docx
