# Supplementary material for: Clusters of diet, physical activity, screen-time and sleep among adolescents and associations with 3-year change in indicators of adiposity
Source: PLoS One. 2024 Dec 23;19(12):e0316186. doi: 10.1371/journal.pone.0316186 (PMC11666017; doi:10.1371/journal.pone.0316186)
Supplement: Attachment — Submitted filename: Response to Reviewers.docx [file pone.0316186.s006.docx]

**Editor Comments:**

| **Editor**  **Point #** | **Editor Comments** | **Response** |
| --- | --- | --- |
| 1 | *The increased reduction in sample size may impact the results' representativeness, generalizability, statistical power, and precision, potentially undermining the validity and trustworthiness of the study's conclusions. Presumably, the initial sample aimed to represent the diversity of the UK population, taking into account socioeconomic, regional, and demographic factors. A relative smaller sample size may limit the capacity to apply the findings to the total UK population, particularly if specific subgroups are underrepresented. Although the authors aimed to prove the sample's representativeness, they did not provide evidence of the representation of all significant subgroups. The investigation of potential confounding variables represents a step forward, but further work is required. To address this issue, the authors should investigate further or acknowledge it as a study limitation.* | Thank you for raising this question.  We have added the below sentence as a limitation in the discussion on page 25, lines 427-429.  *“The analytical sample was representative of the home countries, however the findings may have limited the generalisability to the broader UK population due to the reduced sample size utilised.”* |
| 2 | *The validity and reliability of the questionnaires used to assess screen time and food habits should be developed.* | We have clarified in the methods section (page 6, lines 120-121) that the questionnaires used to assess screen time, food habits and sleep were developed by the cohort management team.  *“This is a secondary data analysis, and all measures (i.e. questionnaires, protocols for device use) were developed by the original cohort team.”*  Also, we have added the below sentence as a limitation in the discussion on page 25, lines 434-437.  *“The validity and reliability of the questionnaires used to assess screen time, food habits and sleep were not independently tested, as they were developed by the cohort management team, which may affect the accuracy of the findings.”* |
| 3 | *In Figure, the data is stacked in a way that makes it difficult to differentiate between categories, as the colors are very similar.* | Thank you for raising this concern regarding the clarity of the figure and the difficulty in differentiating between categories due to the similar colours.  We have edited the colours of all variables to make them more distinct. |
